# The cytotoxic effect of *Baeckea frustescens* extracts in eliminating hypoxic breast cancer cells

**DOI:** 10.1186/s12906-021-03417-9

**Published:** 2021-10-01

**Authors:** S. H. Shahruzaman, F. Z. Yusof, S. Maniam, S. Fakurazi, S. Maniam

**Affiliations:** 1 Department of Human Anatomy, Faculty of Medicine and Health Sciences, Universiti Putra Malaysia, 43400 UPM, Serdang, Selangor Darul Ehsan Malaysia; 2 School of Science, RMIT University, Melbourne, VIC 3001 Australia

**Keywords:** *Baeckea frutescens*, hypoxia, breast cancer, apoptosis, 3-dimensional cell culture

## Abstract

**Background:**

Adaptive metabolic response towards a low oxygen environment is essential to maintain rapid tumour proliferation and progression. The vascular network that surrounds the tumour develops an intermittent hypoxic condition and stimulates hypoxia-inducing factors. *Baeckea frutescens* is used in traditional medicine and known to possess antibacterial and cytoprotective properties. In this study, the cytotoxic effect of *B. frutescens* leaves and branches extracts against hypoxic human breast cancer (MCF-7) was investigated.

**Method:**

The extracts were prepared using Soxhlet apparatus for ethanol and hexane extracts while the water extracts were freeze-dried. *In vitro* cytotoxic activities of *B. frutescens* extracts of various concentrations (20 to 160 μg/mL) at 24, 48, and 72 hours time points were studied using MTT in chemically induced hypoxic condition and in 3-dimensional *in vitro* cell culture system. An initial characterisation of *B. frutescens* extracts was carried out using Fourier-transform Infrared- Attenuated Total Reflection (FTIR-ATR) to determine the presence of functional groups.

**Results:**

All leaf extracts except for water showed IC50 values ranging from 23 -158 μg/mL. Hexane extract showed the lowest IC50 value (23 μg/mL), indicating its potent cytotoxic activity. Among the branch extracts, only the 70% ethanolic extract (B70) showed an IC50 value. The hexane leaf extract tested on 3- dimensional cultured cells showed an IC50 value of 17.2 μg/mL. The FTIR-ATR spectroscopy analysis identified various characteristic peak values with different functional groups such as alcohol, alkenes, alkynes, carbonyl, aromatic rings, ethers, ester, and carboxylic acids. Interestingly, the FTIR-ATR spectra report a complex and unique profile of the hexane extract, which warrants further investigation.

**Conclusion:**

Adaptation of tumour cells to hypoxia significantly contributes to the aggressiveness and chemoresistance of different tumours. The identification of *B. frutescens* and its possible role in eliminating breast cancer cells in hypoxic conditions defines a new role of natural product that can be utilised as an effective agent that regulates metabolic reprogramming in breast cancer.

**Supplementary Information:**

The online version contains supplementary material available at 10.1186/s12906-021-03417-9.

## Introduction

It is estimated that 18.1 million new cases and 9.6 million cancer deaths worldwide in 2018 and breast cancer is being the most commonly diagnosed and leading cause of death among females [1]. Studies have shown 5-10% of newly diagnosed breast cancer patients present with metastasized tumours [2] and the risk of developing metastatic disease following primary tumour resection and adjuvant therapy [3]. Metastases in cancer is the central feature of malignancy and one key causes of failure in cancer therapy [4]. Metastatic progression in tumorigenesis is initiated by genes involved in energy production, angiogenesis, tissue remodelling, enhance cell motility, matrix degradation, and epithelial-to-mesenchymal transition [5]. These genes trigger low aggressive cancer cells to invade surrounding tissues, attract supportive stroma, disperse cancer cells, and infiltrate distant metastatic niches [3]. Hyper-proliferative cancer cells outgrow their surrounding vascular network developing an intermittent hypoxic condition and stimulate hypoxia-inducing factor (HIF) [6].

As cancer progresses, cancer cells exploit and adapt their surroundings to facilitate inappropriate growth, angiogenesis, invasiveness, and ultimately metastasize [7, 8]. The development of a tumour requires an intricate interaction between the spatial organization of the cell surface receptors and the surrounding microenvironment. Cell-based assays have been an important pillar for the drug discovery process and rely on the availability of a versatile platform that is able to recapitulate the pathophysiological features of a native tumour and its microenvironment [9].

All solid tumours contain areas that are well oxygenated, poorly oxygenated, and necrotic cells [10]. The tumour microenvironment can be chemically mimicked or the use *of an in vitro* culture systems that can recapitulate the relevant aspects of solid tumours. Recently, a growing body of evidence has suggested that three-dimensional (3D) culture systems, in contrast to 2D, are more reflective of *in vivo* cellular responses. 3D culture systems have the ability to recapitulate the tumour-like environments. Hence, it provides more accurate and reproducible toxicity information for early stage drug discovery. In this study, the hypoxic environment was mimicked using dimethyloxalylglycine (DMOG), a synthetic analogue of α-ketoglutarate, commonly used to induce HIF signalling and inhibit oxygen consumption in cancer cell lines was used to induce a hypoxic environment [11] as well as 3D culture system.

*Baeckea frutescens* of the family Myrtaceae and subfamily Myrtoideae are found in Malaysia, Indonesia, Southern China, and Australia [12, 13]. The bioactive constituents of *B. frutescens* were shown to possess various properties such as antibacterial [14–16]], antioxidant [17], anti- inflammatory [18] and LDL-oxidation inhibitor in arteriosclerosis [19] as well as some traditional medicine values in treating influenza, dysentery and measles [20].

Our lab has previously explored the cytotoxicity of *B. frutescens* leaves [21] and branch [22] extracts on human breast cancer cells (MCF-7). The extracts showed selective cytotoxicity with IC_50_ of less than 50 μg/mL and inhibited glucose consumption as early as 24 hours after treatment. Paving our way in further exploring the anticancer properties of *B. frutescens,* this study aims to investigate the cytotoxicity effect of *B. frutescens* leaves and branch extracts on hypoxic MCF-7 cells. This is the first study to report on the cytotoxicity effect of *B. frutescens* in hypoxic condition.

## Materials and Methods

### Extracts preparation

*B. frutescens* or Cucur Atap was collected from Forest Research Institute Malaysia (FRIM) Research Station in Setiu, Terengganu upon permission from the local district. Collected plant specimens were authenticated by a botanist, Dr Shamsul Khamis at Institute of Bioscience, Universiti Putra Malaysia. A voucher specimen was deposited with the accession number: KLU 47909 at the herbarium of the Institute of Bioscience, Universiti Putra Malaysia. The leaves and branches of *B. frutescens* were air- dried under shade for 7 days and were pulverized into coarse powder. The coarse powder was extracted using hexane, ethanol, and water as previously described by Shahruzaman et al. [21]. Briefly, for ethanol extracts, 111 g, 142 g, and 200 g of coarse powder were weighed and extracted in 5 l of ethanol to obtain 90, 70, and 50, respectively. A final concentration of 100 μg/ml in the media was used for subsequent experiments. The experimental research conducted in this study was complied with the guidelines from Universiti Putra Malaysia. .

### Cell lines human

MCF-7 breast carcinoma cells (ATCC, USA) were grown in DMEM supplemented with 100 μg/mL streptomycin, 100 IU/mL penicillin, and 10% fetal bovine serum (FBS) (Sigma-Aldrich, USA). Cells were maintained at 37°C in 5% CO_2_.

### Inducing hypoxia

Dimethyloxalylglycine (DMOG), a synthetic analogue of α-ketoglutarate, commonly used to induce HIF signalling and inhibit oxygen (O_2_) consumption in cancer cell lines was used to induce a hypoxic environment [11]. MCF-7 human breast cancer cells were plated into 96-well microplates and was treated with DMOG at various concentration (0 – 2.5 mM) for 72 hours at 37°C in 5% CO_2_.

### Western blot

Cells were lysed with TNN lysis buffer containing protease inhibitor cocktail (leupeptin, pepstatin A, aprotinin, bestatin, AEBSF-HCl, E-64). Equal amounts of proteins were separated by SDS-PAGE and transferred to a nitrocellulose membrane. After blocking, the blots were incubated with primary antibodies HIF-1α (1:1000) (BD Pharmagen, Oxford,UK) and β-actin (1:1000) (Santa Cruz, Heidelberg, Germany). After washing and incubating with mouse secondary antibody (1:10000) (Dako, California, USA), the blots were visualised using enhanced chemiluminescent Western Blotting detection kit (Pierce, ThermoFisher Scientific, USA). The image obtained was analysed using Gene Tools analysis software (Syngene, UK).

### Cell viability assay

MCF-7 human breast cancer cells were plated into 96-well microplates at an initial seeding density of 2 × 10^3^ cells per well in a volume of 200 μl of culture media. Cells were cultured at 37°C in 5% CO_2_ overnight to allow attachment on to the wells. Hypoxic condition was induced with DMOG (Cayman, USA) with a final concentration of 2.0 mM overnight and then treated with increasing concentration (20 to and 160 μg/mL) of *B. frutescens* extracts for 24, 48 and 72 hours.

The MTT assay was conducted as previously described [21]. Briefly, 30 μl of MTT/Phosphate buffered saline (PBS) (Amresco, USA) solution was added into each well and incubated for 4 hours in dark. The formazan grain was dissolved in DMSO (Amresco, USA) and the absorbance which corresponds to viable cells was measured at 570 nm (Wavelength range: 550 – 600 nm) by using an ELISA plate reader (Tecan 200, Switzerland).

### 3-dimensional cells

MCF-7 cells were seeded on VECELL G-plates (Geneion-bio, Malaysia) at a density of 2 × 10^3^ cells and cultured in DMEM supplemented with 10% FBS, 100 μg/mL streptomycin and 100 IU/mL penicillin at 37°C in a humidified atmosphere containing 5% CO_2_. The cells were grown for 72 hours until spheroid were formed (Figure S1). The spheroids were treated with the indicated drugs for 72 hours.

### Fourier transform infrared spectroscopy attenuated total reflection (FTIR-ATR)

FTIR-ATR was used to determine the functional group in the extracts. Nine *B. frutescens* extracts were tested at room temperature in the full scan range between 450–4000 cm^–1^ to determine the functional groups in the extract [23].

### Statistical analysis

Data were expressed as mean ± SEM (Standard Error of the Mean). SPSS (version 20) statistical software was used for the analysis of data. Data were analysed using one way analysis of variance (ANOVA) followed by Bonferroni post-hoc test and p < 0.05 was taken as the level of significance.

## Results and Discussion

Adaptive metabolic response towards a low oxygen environment is essential to maintain rapid tumour proliferation and progression [24]. The vascular network that surrounds the tumour develops an intermittent hypoxic condition and stimulates HIF [25]. HIF is a key regulator responsible for the induction of genes that facilitate the adaptation and survival of cells and the whole organism from normoxia(~ 21% O_2_) to hypoxia (~ 0.5% O_2_) [26]. The stabilization of HIF is a hallmark of hypoxia and results in global transcriptional changes in gene expression, including genes with roles in promoting tumour progression, angiogenesis, metastasis, iron metabolism, glucose metabolism, cell proliferation and survival [27, 28]. Overexpression of HIF-1 has been found in various cancers, and targeting HIF-1 could represent a novel approach to cancer therapy. Adaptation of tumour cells to hypoxia induced HIF-1 significantly contributes to the aggressiveness and chemoresistance of different tumours. HIF-1 plays a central role in tumour pathology and is a target for treatment and therapy [29, 30]. A number of drugs target HIF inhibition which includes EZN-2208 [31], PX-478 [32]. The discovery of HIF-1 has led to an improved understanding of the molecular link between hypoxia and deregulated glucose metabolism [33]. In this study, the hypoxic condition was mimicked by chemically inducing HIF-1 expression or by using a 3D culture system.

**Fig. 1:**
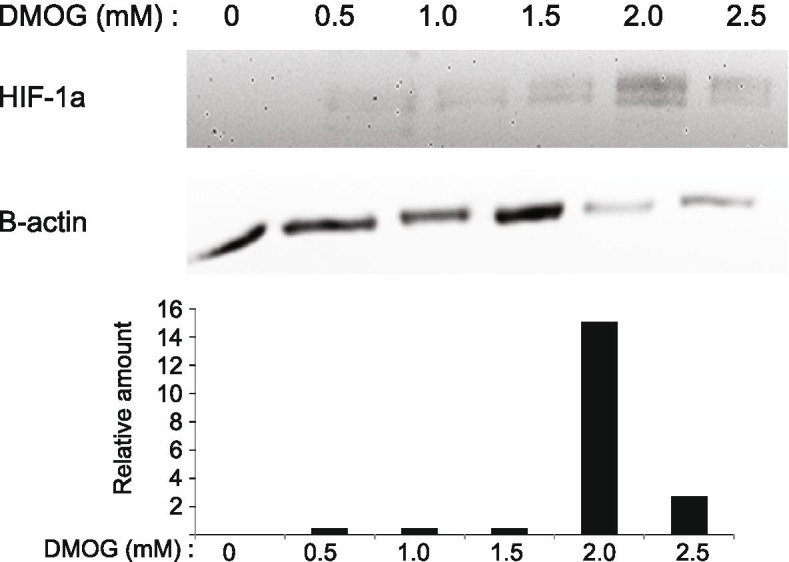
Induction of hypoxic condition using DMOG*.* MCF-7 cells were either treated or untreated with DMOG at various concentrations for 72 hours before harvesting and immunoblotted with antibody as indicated. HIF-1α was detected using HIF-1α antibody. Actin served as loading control. Bar chart shows quantification of HIF-1α levels compared to β-actin control at various DMOG concentrations

In this study, the hypoxic environment was mimicked using dimethyloxalylglycine (DMOG) to induce a hypoxic environment. An initial increase of HIF was observed at 0.5mM. However, at 2.0mM showed an increased HIF expression and this concentration of DMOG was chosen for subsequent experiments (Fig. 1). This method is inexpensive compared with the hypoxic chamber. Hypoxic cancer cells are known to be resistant to radiotherapy and some conventional chemotherapy [34]. The hypoxic microenvironment induces adaptive changes to tumour cell metabolism and can further alter the local microenvironment [35].

The methanolic derivatives from *B. frutescens* were shown to exhibit cytotoxic activity against leukaemic cells [36], human lung, pancreatic, and breast cancer cells [15]. Previously, we have measured the cytotoxic activity of hexane, water and ethanolic *B. frutescens* extracts (0-160 μg/ml) from leaves against MCF-7 cells for 24, 48 and 72 h in normoxic condition. It was noted that hexane extract of *B. frutescens* leaves possess selective cytotoxic activity and modulate glucose uptake in breast cancer cells [21, 22]. To our knowledge, this is the first report describing the cytotoxic activity of *B. frutescens* extracts against MCF-7 cells in hypoxic condition.

**Fig. 2:**
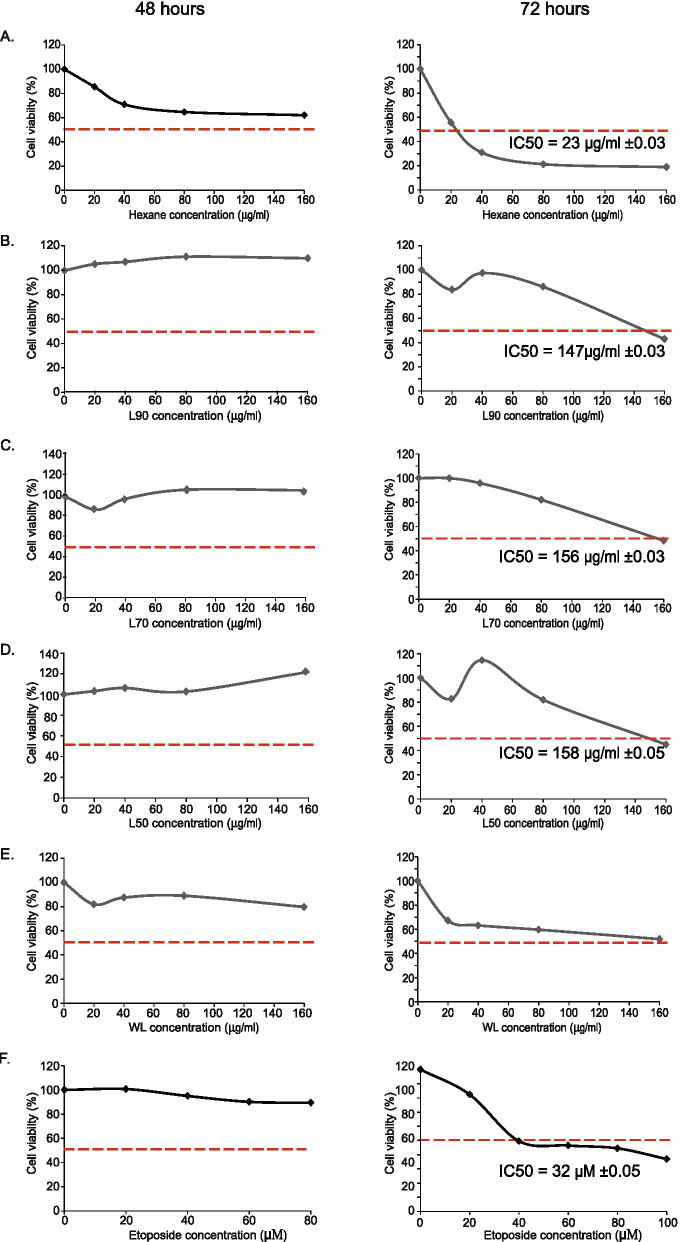
Effects of *B. frutescens* leaves extracts on MCF-7 cell viability in hypoxic condition*.* Cytotoxicity was determined using cell viability assay and the IC50 value calculated as half maximal percentage of cell viability inhibition is indicated by the red horizontal line. Hypoxic condition was induced in MCF-7 cells with 2.0 mM of DMOG and incubated with either (**a**) hexane, (**b**) L90, (**c**) L70,(**d**) L50 or (**e**) water extract of *B. frutescens* leaves or (**f**) etoposide, which served as positive control for 48 (left panels) or 72 (right panels) hours. Data is expressed as mean ± standard error mean based on six independent experiments with triplicate wells for each concentration

**Fig. 3:**
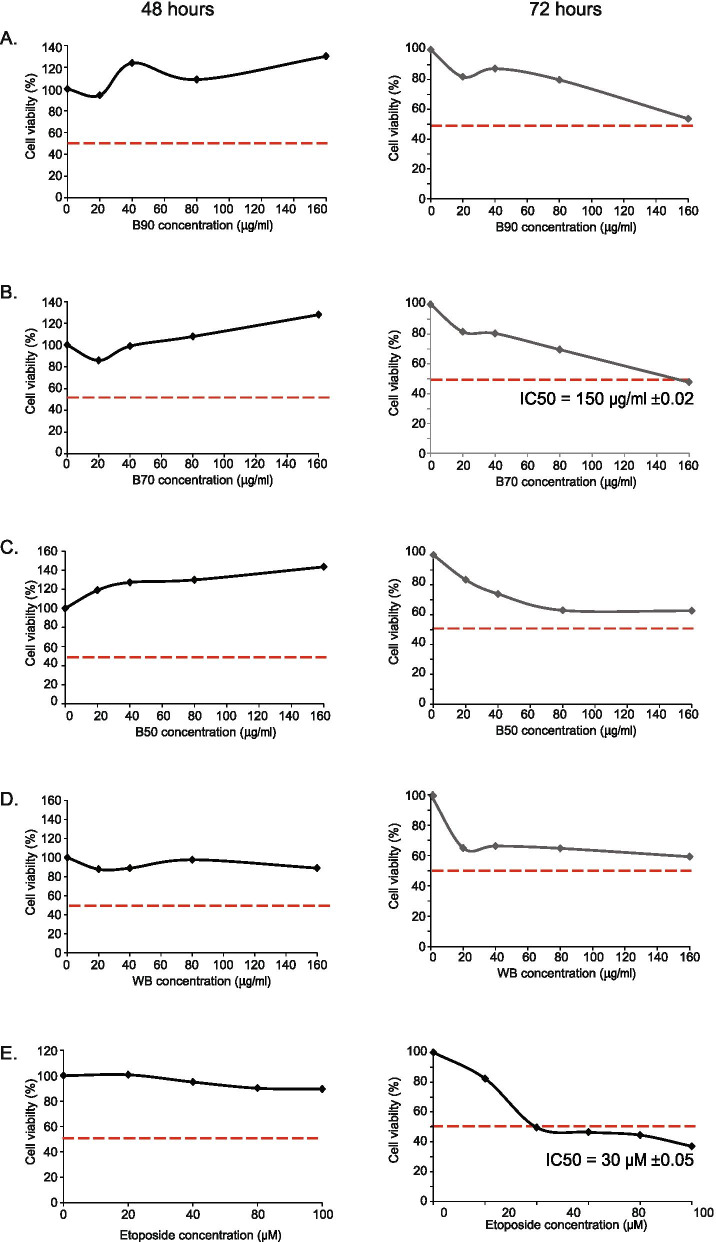
Effects of *B. frutescens* branches extracts on MCF-7 cell viability in hypoxic condition. Cytotoxicity was determined using cell viability assay and the IC50 value calculated as half maximal percentage of cell viability inhibition is indicated by the red horizontal line. Hypoxic condition was induced in MCF-7 cells with 2.0 mM of DMOG and incubated with either (**a**) WB, (**b**) B90, (**c**) B70 or (**d**) B50 of *B. frutescens* branches or (f) etoposide, which served as positive control for 48 (left panels) or 72 (right panels) hours. Data is expressed as mean ± standard error mean based on six independent experiments with triplicate wells for each concentration

**Fig. 4:**
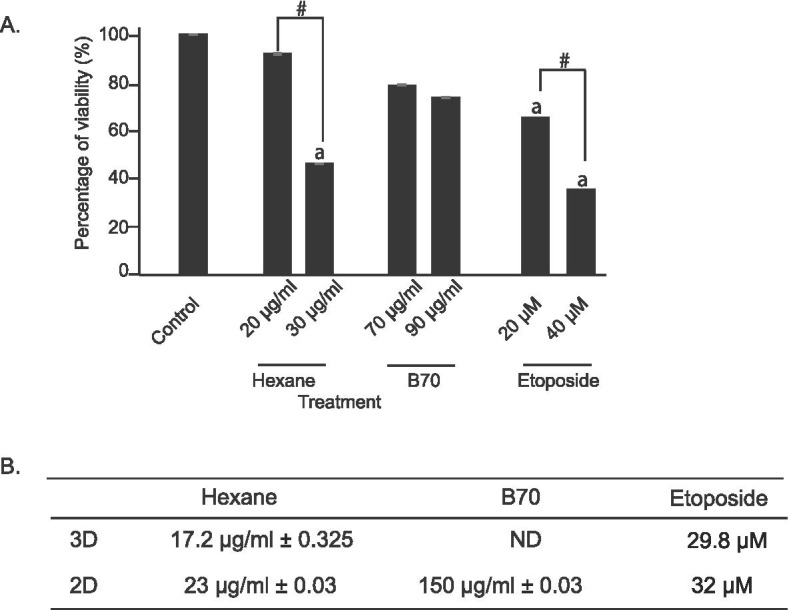
Effects of selective *B. frutescens* extracts on 3-Dimensional cell viability. Cytotoxicity was determined using cell viability assay and the IC50 value calculated using 3D culture system. MCF-7 cells were either untreated or treated with hexane, B70 or etoposide for 72 hours at indicated concentrations (**A**). Etoposide served as positive control and DMSO was used as vehicle control. IC50 value obtained from the 3D *in vitro* system and chemically induced hypoxic condition (2D) were compared (**B**). p<0.05. a indicate statistically significant compared to control and # indicate statistically significant within group. Data are displayed as mean ± SD

Cytotoxic activities of nine *B. frutescens* extracts from leaves and branches were tested against MCF-7 cell lines for 24, 48, and 72 hours in hypoxic condition. Etoposide was used as a positive control. After 24 hours (data not shown) and 48 h of treatment, IC_50_ values of these extracts were unable to determine (left panel, Figs. 2 and 3). However, after 72 hours of treatment, all *B. frutescens* leaves extracts except WL showed cytotoxic activity (Fig. 2). The IC_50_ values of L90, L70 and L50 were higher than 100 μg/mL. Hexane extract has the most potent with IC_50_ value of 23 μg/mL. Out of the four branch extracts tested, only B70 showed cytotoxic activity with IC_50_ value of 150 μg/mL (Fig. 3). The IC_50_ values of *B. frutescens* extracts in hypoxic conditions were indicated in Figs. 2 and 3.One potent extract from the branches (B70) and leaves (hexane) were chosen to be tested in the 3D *in vitro* culture system. Growing body of evidence has suggested that 3D culture systems represent more accurately of the actual tumour microenvironment. Hence, the 3D model-based assays serve as one of the key tools to assess the potential efficacy of these compounds. Hexane extract at its highest concentration and etoposide at all concentration showed significantly lower cell viability compared to the vehicle control in the 3D *in vitro* system. No significant changes were observed in B70 (Fig. 4a).

Both hexane and etoposide showed lower IC_50_ value in the 3D *in vitro* system compared to the chemically induced hypoxia (Fig. 4b). All solid tumours contain areas that are well oxygenated, poorly oxygenated, and necrotic cells [10]. The tumour microenvironment can be chemically mimicked or the use *of an in vitro* culture systems that can recapitulate the relevant aspects of solid tumours. The use of 3D culture systems in contrast to 2D were shown to be more reflective of *in vivo* cellular responses. 3D culture systems have the ability to recapitulate a tumour-like environment. Hence, it provides more accurate and reproducible toxicity information for early stage drug discovery.

Interestingly, the IC_50_ value obtained in normoxic condition (17 μg/mL) as previously reported [21] were much lower compared to the values obtained in hypoxic condition. Furthermore, it was noted that IC_50_ of the hexane extract in normoxic condition was obtained as early as 48 hours [21] which is a shorter duration compared to the IC_50_ obtained in hypoxic condition (72 hours). This is most likely due to aggressive growth stimulated by hypoxic conditions, hence a higher amount of extract and longer time are required to suppress cell viability. A typical tumour microenvironment consists of fibroblasts, myofibroblasts, neuroendocrine cells, adipose cells, immune and inflammatory cells, blood and lymphatic vascular networks, and the extracellular matrix [37]. The lack of oxygenation results in increased collagen deposition or also known as desmoplasia [38]. The dynamic function of desmoplastic stroma during tumorigenesis that supports tumour growth, progression, and recruitment, through other cellular and noncellular constituents evolves with the genetic profile changes of cancer cells [39, 40]. Adaptation of cancer cells to a hypoxic condition leads to a more aggressive and therapeutically resistant tumour phenotype [6].

**Fig. 5:**
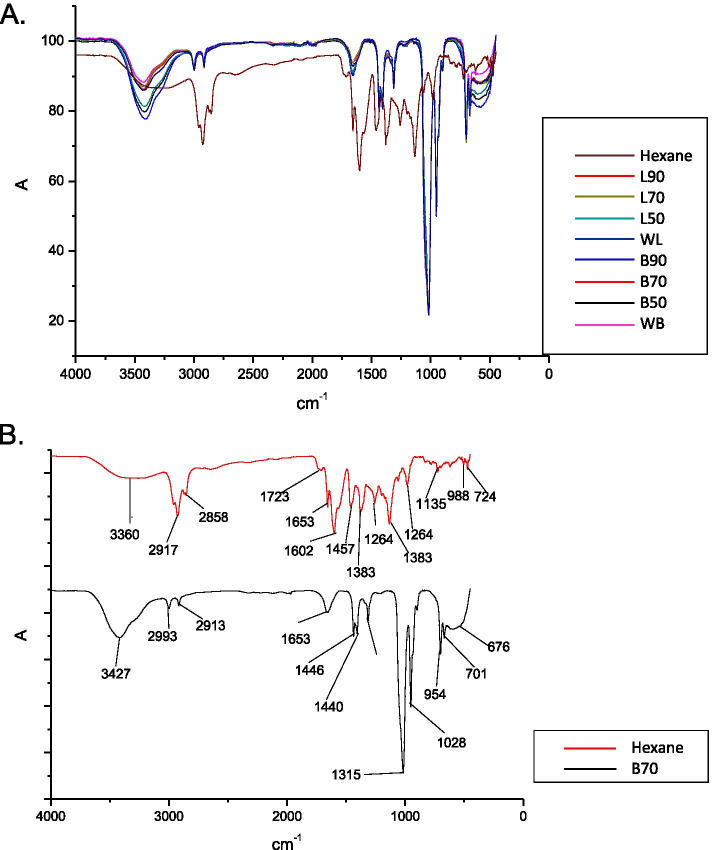
FTIR-ATR spectra of *B. frutescens* extracts in the range of 450-4000 cm ^−1^. Combined FTIR spectra of *B. frutescens* leaves and branches extracts (**A**). FTIR spectra of *B. frutescens* leaves(hexane) and branches extracts; B70 (**B**)

The initial characterisation of *B. frutescens* extracts of leaves and branches were carried out using FTIR- ATR to determine the presence of functional groups (Fig. 5). Taking into account that *B. frutescens* leaves and branch extracts are a combination of several molecules, their infrared spectra are complex and include a variety of bands from various functional groups. Similar spectra were obtained for the polar extracts of WB, WL, L70, L50, B50, B90 and B70. Taking B70 as an example, an intense signal at 3426 cm^-1^ characteristic for the stretching of O-H bond and a weak signal at 2995-2911 cm-1, which corresponds to C-H bond stretching were observed. In the fingerprint region, a weak signal 1654 cm^-1^ which were assigned to C=C or C=O stretching and the strong signal at 1315 cm^-1^ were attributed to the C-O stretching. These results indicate the characteristic presence of glycosides, several different kinds of carbonyl compounds, and flavonoids (Table 1) [20]. In the nonpolar extract, hexane, intense signals at 2917-2856 cm^-1^ which correspond to C-H stretching of saturated hydrocarbons were recorded. Additionally, strong signals at 1653, 1602, and 1457 cm^-1^ attributed to C=C and aromatic ring skeletal stretching were also observed. This extract has a significantly different infrared spectrum profile compared to the other polar extracts and has not been previously reported. Hexane being a nonpolar solvent, extracts lipophilic compounds which are known to have better uptake and absorption rate in cells compared to polar compounds. FTIR-ATR spectroscopy analysis of the hexane extract suggests that large lipophilic groups such as aliphatic chain and aromatic structures are part of the compound with no significant polar functional groups.

**Table 1:** Assignment of FTIR signals for *B. frutescens* leaves and branches extracts in hexane, H and B70

Frequency (cm^**−1**^)		Type of bond	Functional group
HX	B70		
	3426	O–H	Alcohol
2917, 2858	2995, 2911	C-H, C≡C	Alkene, Alkyne
1653,1602	1654	C=O, C=C	Alkene, carbonyl
1457, 1383	1447,1441	C=C	Aromatic rings
	1315	C-O	Ether, ester or carboxylic acid
	1028	C–OH	Secondary alcohol functional group
	956	C–OH	Primary alcohol functional group

## Conclusion

In conclusion, this study reports *B. frutescens* extracts of both branch (B70) and leaf (hexane, L90, L70, and L90) extracts are able to induce cell death in hypoxic condition. Hexane extract from the leaves was shown to induce cell death in both chemically induced hypoxic condition as well as 3D *in vitro* culture system. In DMOG-induced hypoxic condition, *B. frutescens* extracts require longer treatment time and higher concentrations to produce cytotoxic effect compared to normoxic condition. The FTIR-ATR spectroscopy analysis identified various characteristic peak values with different functional groups attributed to compounds, mainly flavonoids, chromones, branched hydrocarbons, and phloroglucinols. Further experiments are required to characterise the FTIR-ATR profile of the hexane extract. Following our previous reports, the findings in this study reiterate the potential of *B. frutescens* as an anticancer agent for breast cancer.

## Supplementary Information

**Additional file 1: Figure S1**. Morphology of cells in 3-dimesional culture system. MCF-7 cells were cultured on VECELL G-plate for 72 hours until spheroid were observed.

## Data Availability

The datasets analysed during the current study are available from the corresponding author on reasonable request.

## Abbreviations

3D: 3-dimensional
B50: *B. frutescens* branches prepared at the ratio of 1:50 in ethanol B70 *B. frutescens* branches prepared at the ratio of 1:70 in ethanol B90 *B. frutescens* branches prepared at the ratio of 1:90 in ethanol DMOG Dimethyloxalylglycine
DMEM: Dulbecco’s Modified Eagle Medium DMSO Dimethyl sulfoxide
EGF: Epidermal growth factor
ER: Oestrogen receptor
FTIR-ATR: Fourier transform infrared spectroscopy attenuated total reflection HIF Hypoxia-inducing factor
IC50: Half maximal inhibitory concentration
L50: *B. frutescens* leaves were prepared at the ratio of 1:50 in ethanol L70 *B. frutescens* leaves were prepared at the ratio of 1:70 in ethanol L90 *B. frutescens* leaves were prepared at the ratio of 1:90 in ethanol MTT 3-(4,5-dimethylthiazol-2-yl)-2,5-diphenyltetrazolium bromide PBS Phosphate-buffered saline
WB: - *B. frutescens* branch water extract
