# Supplementary material for: The cytotoxic effect of Baeckea frustescens extracts in eliminating hypoxic breast cancer cells
Source: BMC Complement Med Ther. 2021 Oct 1;21:245. doi: 10.1186/s12906-021-03417-9 (PMC8485548; doi:10.1186/s12906-021-03417-9)
Supplement: Supplementary file 1 — Additional file 1: Figure S1. Morphology of cells in 3-dimesional culture system. MCF-7 cells were cultured on VECELL G-plate for 72 hours until spheroid were observed. [file 12906_2021_3417_MOESM1_ESM.docx]

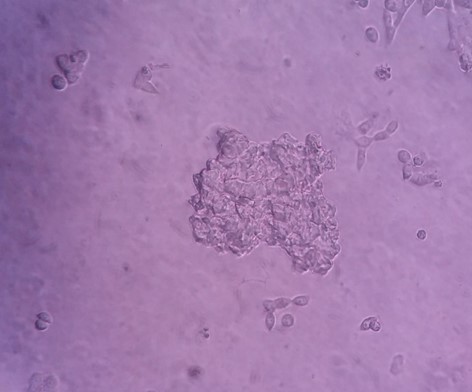


# Figure S1 – Morphology of cells in 3-dimesional culture system

MCF-7 cells were cultured on VECELL G-plate for 72 hours until spheroid were observed.
